# Supplementary material for: Human Adipose-Derived Stromal Cells Delivered on Decellularized Muscle Improve Muscle Regeneration and Regulate RAGE and P38 MAPK
Source: Bioengineering (Basel). 2022 Aug 30;9(9):426. doi: 10.3390/bioengineering9090426 (PMC9495328; doi:10.3390/bioengineering9090426)
Supplement: Supplementary file 1 [file bioengineering-09-00426-s001.zip › bioengineering-1886031-supplementary.pdf]

Table S1. Nanostring analysis of differentially expressed genes between Sham v. DMM and Sham v. Empty Defect. Genes shown are associated with a p-value of 0.05 or less.

DMM Upregulated Genes	DMM Downregulated Genes	Empty Upregulated Genes	Empty Downregulated Genes
Ackr1	Gtf2ird1	Acin1	Dlat
F2	Nova1	Angpt2	Dot1
Gria2		Ap3s1	
Sf3b2		Apoe	
Tbp		App	
		Arsa	
		Atp6v0e1	
		Atp6v1a	
		Bax	
		Cacna1a	
		Casp3	
		Casp6	
		Cers6	
		Col6A1	
		Ap	
		Ctse	
		Ep300	
		Erbb3	
		Erg	
		Fn1	
		Fus	
		Gnai2	
		Gnai3	
		Gng2	
		Grik2	
		Grn	
		Gss	

		Gtf2b	
		Gusb	
		Hdac1	
		Hgf	
		Hif1a	
		Hmox1	
		Htra2	
		Idh1	
		Itga5	
		Itpr3	
		Jam	
		Jun	
		Lama2	
		Lama5	
		Lamb2	
		Lmna	
		Lrp1	
		Mapk3	
		Mgmt	
		Mmp2	
		Myc	
		Myh8	
		Naglu	
		Nf32l2	
		Nr4a2	
		Nsf	
		Ntf3	
		P2ry12	
		Palm	
		Pde1b	
		Pik3r1	
		Pla2g4a	
		Plcb1	

		Plcb4	
		Plekho2	
		Prkcsh	
		Psen2	
		Ptdss1	
		Rhoa	
		Rras	
		Runx-2	
		Scamp2	
		Sirt7	
		Slc11a1	
		Spp1	
		Src	
		Sri	
		Stat3	
		Stx2	
		Synj1	
		Tcirg1	
		Tgfb1	
		Tgfbr2	
		Trim28	
		Trim37	
		Trpm2	
		Tspo	
		Ugcg	
		Xab2	
		Cnot10	
